# Evaluation of the immune status of birds and domestic and companion animals for the influenza A virus in Eastern Saudi Arabia

**DOI:** 10.14202/vetworld.2020.1966-1969

**Published:** 2020-09-23

**Authors:** Abdelmohsen Abduallah Alnaeem, Abdulkareem Al-Shabeb, Maged Gomaa Hemida

**Affiliations:** 1 Department of Clinical Studies, College of Veterinary Medicine, King Faisal University, Saudi Arabia; 2 Department of Anatomy, College of Veterinary Medicine, King Faisal University, Al-Hasa, Saudi Arabia; 3 Department of Microbiology, College of Veterinary Medicine, King Faisal University, Saudi Arabia; 4 Department of Virology, Faculty of Veterinary Medicine, Kafrelsheikh University, Egypt

**Keywords:** enzyme-linked immunosorbent assay, influenza virus, livestock, Saudi Arabia, serology, type A

## Abstract

**Background and Aim:**

Influenza type A virus infections are still one of the major concerns for the health of humans and various species of domestic and companion animals. Wild birds play an essential role in the transmission cycle of the virus. Regularly monitoring the spread of the virus is a significant step in its mitigation. Highly pathogenic avian influenza viruses, including H5N1 and H5N8, have been reported in birds in the Arabian Peninsula, including Saudi Arabia, in recent decades. This study aimed to evaluate the immune status of birds, domestic and companion animals for Influenza type A virus in Eastern Province of Saudi Arabia.

**Materials and Methods:**

We collected 195 serum samples from dromedary camels, sheep, goats, native breed chickens, doves, dogs, and cats. We tested these sera for the presence of specific antibodies against influenza type A virus using a commercially available enzyme-linked immunosorbent assay.

**Results:**

Our results show that 4% of the tested samples had antibodies in sera, including some doves, chickens, and dogs. These data suggest exposure and seroconversion of these animals or birds to the influenza type A virus.

**Conclusion:**

The presence of antibodies against influenza type A virus in sera of some animals and birds without a previous vaccination history against the virus indicates a natural exposure history regarding this virus and seroconversion. Further large-scale molecular and epidemiological studies are needed to obtain a better understanding of the dynamics of influenza type A virus among various species of animals and birds.

## Introduction

Respiratory viral infections represent major concerns to human and animal health. The influenza virus is one of the common respiratory pathogens of humans and animals. The influenza virus belongs to the *Orthomyxoviridae* family and the *Alphainfluenzavirus* genus. There are four types of influenza viruses (A, B, C, and D). Influenza type A virus causes disease in a wide range of animals, birds, and humans. Birds are susceptible to all types of influenza type A viruses. It is host-specific, but the occasional transmission of the virus between different species has induced several outbreaks. Avian and swine are considered the most important sources for the transmission of influenza type A virus to humans [1]. Accumulating evidence exists regarding the zoonotic transmission of H5 and H7 strains originating from birds. In most cases, infection may be asymptomatic or induce very subtle clinical symptoms [2,3]. However, severe disease with a case fatality rate of up to 40% has also been reported [4]. In most cases, the transmission is more likely in regions with a high prevalence of avian influenza viruses (AIVs) and the presence of live animal markets [1]. In the case of dogs, evidence of infection with H1N1, H3N2, H3N8, and H9N2 strains has been reported [5-7]. A severe outbreak of respiratory illness among racing dogs caused by H3N8 equine influenza virus from horses that lived near these dogs was reported [8]. There is no evidence for the transmission of influenza A virus from dogs to humans. In the case of cats, sporadic interspecies transmission with avian [9], canine [6,10], human [11], and equine [12] carriers has previously been reported. An outbreak of influenza viruses circulating in felines has the potential to play a role in generating a pandemic of AIV strains and may infect humans. Compared to dogs, evidence of the potential roles of cats as a source of influenza A virus transfer to humans has been previously suggested [1]. This was documented by a human infection during an intense exposure to infected cats. These cats were affected by an outbreak caused by avian influenza A (H7N2) [13]. However, the roles of various animals living in close proximity to humans and regarding the transmission of various types of influenza viruses should not be underestimated.

This study shows that some native Saudi chicken breeds, certain doves, and dogs seroconverted for the influenza type A virus. A large-scale influenza type A virus seroprevalence study should be implemented to include a large number of domestic animals and birds in addition to other companion animals. This will assist in fine-tuning the vaccination programs against influenza viruses, particularly avian influenza, in the future.

This study aimed to evaluate the immune status of birds, domestic and companion animals for Influenza type A virus in Eastern Province of Saudi Arabia.

## Materials and Methods

### Ethical approval

We conducted this study according to the Guidelines of the Animal Ethics Protocols and the National Committee of Bio-Ethics, King Abdul-Aziz City of Science and Technology, Royal Decree No. M/59 (http://www.kfsh.med.sa/KFSH_WebSite/usersuploadedfiles%5CNCBE%20Regulations%20ENGLISH.pdf).

### Collection of serum samples

A total of 169 serum samples were collected from animals, such as dromedary camels (5), sheep (17), goats, dogs (16), and cats (30), and birds such as native breed chickens (54), seagulls (3), pigeons (26), and doves (22).

The selection of animals and birds was based on several criteria, and all sampled animals and birds were healthy, with no signs of diseases, especially respiratory signs. We included various species of domestic animals, such as cattle, sheep, goats, and native breed chickens. This was in addition to some companion animals, such as dogs and cats. In addition, we included certain birds used that shared habitats or came close by the domestic animal and bird samples in these studies, which may have zoonotic potential for AIV. None of the animals and birds were vaccinated against influenza viruses.

Samples were collected during the fall and winter seasons from September 2018 to March 2019. Samples were collected from the Eastern Province of Saudi Arabia. Samples were collected from the target vein per each species of animals, including the jugular vein in the case of dromedary camels, sheep, goats and Sea gale. Serum samples were collected from the saphenous vein from dogs and cats, while samples were collected from the wing veins from chickens and doves. Serum samples were collected in tubes containing anticoagulants. The blood samples were centrifuged at 5000 rpm for 10 min. The separated sera were stored at −20°C for further testing.

### Vaccination and management practices

All sampled animals and birds were unvaccinated against common viral diseases, especially the AIV. Domestic animals, such as cattle, sheep, goats, camels, and chickens, were reared at the King Faisal Agriculture and Veterinary Station. Animals were housed in separate compartments and supplied with food, water, shelter, and veterinary care.

### Enzyme-linked immunosorbent assay (ELISA)

Serum samples were heat inactivated at 56°C for 30 min. We used ID Screen® Influenza A Antibody Competition Multi-species (catalog no: FLUACA-5P) for the analysis. We conducted an ELISA procedure per the manufacturer’s instructions and as previously described [14,15].

## Results and Discussion

A total of 169 serum samples were collected from 10 species of animals and tested for the presence of influenza type A antibodies. Among the species tested, positive results were detected only in chicken, doves, and dogs with an overall prevalence of 4% from the 169 tested serum samples. Results show that 11% of the 45 tested chicken sera and 4% of the 22 tested dove sera were positive. The serum from only one dog was available for testing and was positive. None of the sheep, goat, and seagull sera were positive (Table-1). Among influenza viruses, influenza type A virus constitutes a substantial threat for both human and animal population. Humans are primarily affected by H1N1 and H3N2 subtypes. Occasional pandemics in humans as a result of pigs and birds have been reported [16]. In our study, among the five tested bird species, a seroprevalence of 11% against influenza type A virus was detected in chicken. Several outbreaks of H5N1 highly pathogenic avian influenza in KSA have been reported in poultry and ostrich [17]. Pigeons and doves are frequently seen mingling within close proximity of human accommodations. In our study, one dove (4.5%) tested positive, while all pigeons were negative. The emergence of a pandemic caused by HP-AIV prompted the study of the role of pigeons and doves as an intermediate host of HP-AIV transmission from poultry to humans. In summarizing 32 studies, an average seroprevalence of 8.01% in pigeons and doves was estimated. However, their role as an intermediate host of HP-AIV from poultry to humans was inconclusive [18]. Some companion animals, such as dogs and cats, are known to be infected by various influenza subtypes [8,19]. We tested 30 cats and 1 dog sera, and only the dog sera tested positive. A study conducted in Poland revealed that the seroprevalence of 2.21% of tested dogs was positive for influenza type A virus [20]. However, in Italy, none of the dog or cat sera tested positive during the concurrent circulation of AIV subtypes [21]. Recently, increasing attention has been directed to dromedary camels as a potential reservoir of zoonotic diseases, especially the Middle East respiratory syndrome coronavirus (MERS-CoV) [22]. For instance, dromedary camels are the only known, so far, reservoir for MERS-CoV in humans [23]. In our study, none of the local camels were seropositive for the influenza type A virus. However, we only tested five samples; thus, more studies and a larger number of samples are needed. In KSA, molecular surveillance of influenza type A virus showed that only 1.7% of the imported camels from Africa were positive [22]. Another study showed positive serological results for both H1N1 and H3N2 viruses in the sera of camels from Nigeria [24]. The results of this study have limitations since some species were not represented by an adequate number of samples. Pigs and birds are a proven source of influenza type A infection in humans. However, the roles of other mammals and birds should not be overlooked. It is of significant importance that the vigilant serological and molecular surveillance of influenza type A virus, in various animal species, becomes a routine practice as a fulfillment of the one health concept.

**Table-1:** Results of the serosurveillance of influenza type A virus in some animals and birds in Eastern Saudi Arabia.

Species	Camel	Sheep	Goats	Sea gale	Pigeon	Dove	Cat	Dog	Chicken	Total
**Tested**	5	17	16	3	26	22	30	1	54	195
(+Ve)	0	0	0	0	0	1	0	1	6	8
(−Ve)	5	17	16	3	26	21	30	0	41	169

## Conclusion

This study provides evidence of previous exposure to influenza type A virus in chickens, doves, and dogs in Eastern Saudi Arabia. The presence of specific antibodies against influenza type A in some species of animals and birds (doves, native breed chickens, and dogs), which were unvaccinated against influenza viruses, indicates their exposure to natural viral infection.

## Authors’ Contributions

MGH, AA, and AAA collected specimens, performed the laboratory techniques, data analysis, and wrote the manuscript. All authors read and approved the final manuscript.
